# Proper management of suspicious actinic cheilitis

**DOI:** 10.1186/s40902-019-0198-0

**Published:** 2019-04-09

**Authors:** Soung Min Kim, Hoon Myoung, Mi Young Eo, Yun Ju Cho, Suk Keun Lee

**Affiliations:** 1 Oral and Maxillofacial Microvascular Reconstruction LAB, Brong Ahafo Regional Hospital, Sunyani, Ghana; 2 Department of Oral and Maxillofacial Surgery, Dental Research Institute, School of Dentistry, Seoul National University, 101 Daehak-ro, Jongno-gu, Seoul, 110-768 South Korea; 3 Department of Oral Pathology, College of Dentistry, Gangneung-Wonju National University, Gangneung, South Korea

**Keywords:** Actinic cheilitis (AC), Cytokeratin immunostaining, Epimyoepithelial carcinoma (EC), Lip cancer, Minor salivary gland (MSG)

## Abstract

**Background:**

Actinic cheilitis (AC) is a variant of actinic keratosis which is known to be a premalignant condition that could develop into squamous cell carcinoma (SCC). Epimyoepithelial carcinoma (EC) is a very rare salivary gland (SG) neoplasm that has classical biphasic histologic findings of small tubules and glandular lumina surrounded by clear myoepithelial cells.

**Case presentation:**

We report a very rare case of AC occurring on the lower lip of a 70-year-old woman, which is developing to the EC later.

**Conclusions:**

Diverse appearances of AC include edematous reddish in the acute stage and grey-whitish or dried hyperkeratotic wrinkled lesions in the chronic stage for several months or even years. Accurate treatment of AC in its initial stage could be recommended to avoid further malignant transformation; proper management of clinically suspicious AC is suggested.

## Background

Actinic cheilitis (AC), also termed actinic cheilosis, actinic keratosis of the lip, solar cheilosis, sailor’s lip, and farmer’s lip, is a type of lip inflammation caused by long-term sunlight exposure. This burn-resembling disease is a variant of actinic keratosis, which occurs on the lip [1], and is known to be a premalignant condition that could develop into squamous cell carcinoma (SCC) [1, 2].

Epimyoepithelial carcinoma (EC), previously called epithelial myoepithelial carcinoma (EMC), is a very rare salivary gland (SG) neoplasm that has classical biphasic histologic findings of small tubules and glandular lumina surrounded by clear myoepithelial cells. This uncommon low-grade neoplasm accounts for 1–2% of SG tumors arising in the intercalated ducts, is most commonly found in the parotid gland at more than 85% incidence, and has female preference with the peak age of 60 to 70 years [3, 4].

EC can be diagnosed with myoepithelial and epithelial components of intercalated ducts through immunohistochemistry. Here, we report a rare case of EC in the lower lip that developed from AC over the course of 3 years and suggest the proper management of AC with related literature review.

## Case presentation

**Fig. 1:**
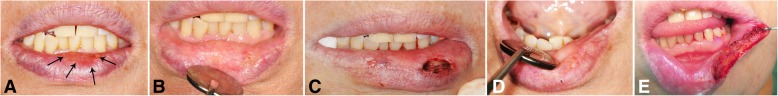
A 70-year-old Korean female exhibited a reticulated red plaque on her lower lip (**a**), disappearance after corticosteroid therapy for 3 months (**b**), re-appearance on the lateral side from its original location in an excisional biopsy state (**c**), whitish plaque after 6 months (**d**), and V-shaped wedge resection after malignancy confirmation (**e**)

A 70-year-old Korean female was referred to our oral and maxillofacial department with recurrent keratosis in the lower lip over the course of 3 years (Fig. 1a). She was diagnosed with oral lichen planus (OLP) in another hospital 2 years prior and received a corticosteroid application without complete symptom relief.

The patient looked healthy without any other skin or oral mucosal diseases and had her natural dentition without any removal dentures or prosthetics replacing her anterior teeth on both the maxilla and mandible. The patient also had no history of smoking, alcohol consumption, or hospitalization. Her occupation over the past 30 years involved selling crabs in a large fish market; thus, she often smelled fresh crabs and tasted marinated and seasoned crabs.

The hyperkeratotic white plaque lesion was round and superficial in the lower middle lip site (Fig. 1a). The patient desired surgical examination after its location altered to the lateral side (Fig. 1b). A superficial excisional biopsy (Fig. 1c) was performed, and an initial stage of SCC was revealed. We hypothesized that OLP transformed into malignancy due to chronic irritation of her lower lip. Therefore, additional cancer work-ups such as supplemental images like computed tomography (CT), magnetic resonance imaging (MRI), and positron emission tomography-computed tomography (PET-CT) were obtained. No metastasis, significant hypermetabolic lesion in the neck, or remaining suspicious lesions were observed in these examinations.

After the first excisional biopsy, a keratotic whitish plaque lesion suspicious of recurrence appeared on the patient 6 months later (Fig. 1d). After having a consent form for operation, we performed a wide V-shaped wedge resection (Fig. 1e). Superficial lip mucosa with underlying orbicularis oris muscles were excised with a 5.0-mm safety margin on the lip surface, and direct closure with layered sutures was performed after negative margin confirmation in frozen biopsy.

The specimen was sent to the Department of Oral Pathology at GangneungWonju National University Dental Hospital and fixed, embedded with paraffin, and microsectioned at 4-μm thickness for pathologic diagnosis. The microsections were routinely stained with hematoxylin and eosin and observed under ordinary light microscopy (U-POT®, Olympus Co., Japan). The microscopic images were captured by a digital camera (DP-70®, Olympus Co., Japan) and analyzed for the article submission under the approval of the Institutional Review Board of Seoul National University (S-D2017006).

**Fig. 2:**
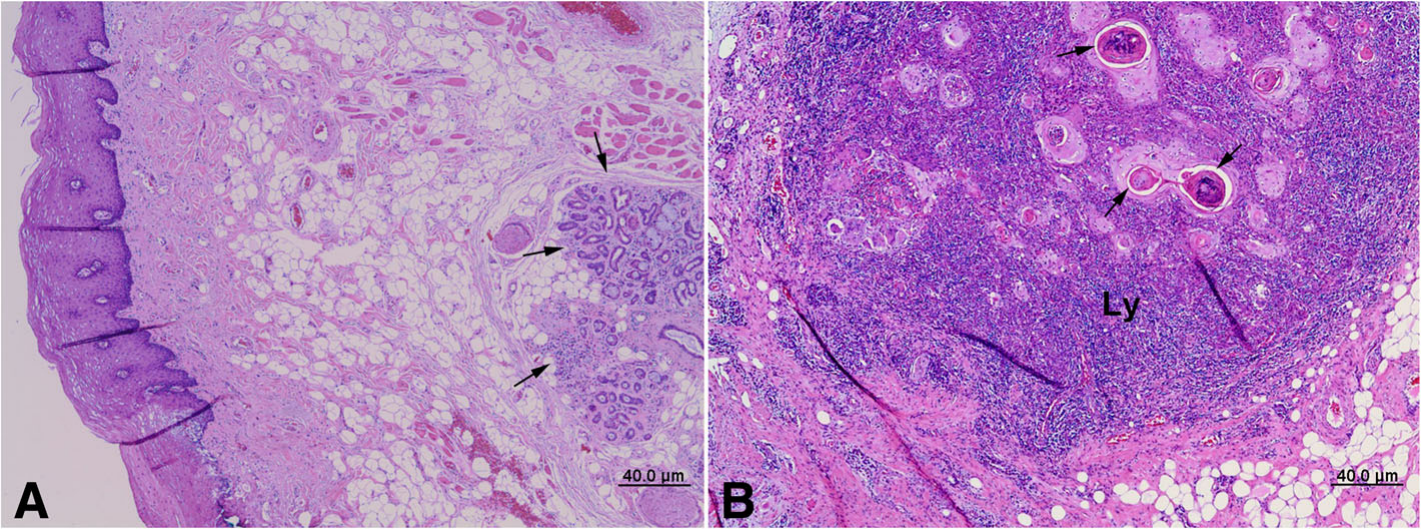
Photomicrographs of epimyoepithelial carcinoma from the lower lip in hematoxylin and eosin stain, normal architecture of mucosa epithelium and fibromuscular adipose tissue containing a minor salivary gland with marked ductal hyperplasia and inflammatory cell infiltration (arrows) (**a**), severely keratinized and exhibited comedo-type necrosis and luminal sequestration of the keratinized epithelium, mimicking glandular duct structures (arrows) and relatively well-localized and typically surrounded by abundant lymphoid tissue (Ly) (**b**)

The microsection exhibited normal architecture of lip mucosal epithelium and fibromuscular adipose tissue containing minor salivary glands (MSGs). The MSGs showed marked ductal hyperplasia with inflammatory cell infiltration. The epithelial tumor became severely keratinized and exhibited comedo-type necrosis and luminal sequestration of the keratinized epithelium mimicking the glandular duct structure, and the tumor cells were relatively well-localized and typically surrounded by abundant lymphoid tissue (Fig. 2). Some areas of keratinized tumor epithelium showed the typical features of epimyoepithelial islets seen in Mikulicz disease, and some tumor epithelium formed pseudo-ductal structures with active lymphocytic reactions under high magnification.

**Fig. 3:**
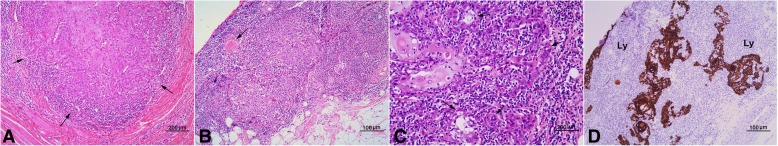
The well-differentiated epithelial tumor cells with highly keratinization in the tumor cluster, proliferation through budding and branching (arrows), similar to glandular ductal growth in the periphery (**a**), epimyoepithelial islets found in Mikulicz Disease in some keratinized tumor epithelium (arrow) (**b**), pseudo-ductal structures (arrows) with active lymphocytic reactions in some tumor epithelium (**c**), and keratinized tumor epithelium to be floating in the lymphoid stroma (Ly) with no features of infiltrative growth into adjacent fibromuscular adipose tissue in cytokeratin immunostaining (**d**)

The pathologic lesion was confined to the vermilion border without the involvement of the oral mucosa or orbicularis oris muscle with a 2-mm lesion depth. In the periphery region, the tumor cells did not grow invasively, but proliferated in a budding and branching fashion similar to glandular ductal growth. In cytokeratin immunostaining, the keratinized tumor epithelium seemed to float in the lymphoid stroma with no feature of infiltrative growth into adjacent fibromuscular adipose tissue (Fig. 3). Therefore, this lesion was finally diagnosed as lower lip EC originating from AC. The patient was instructed to avoid any trauma to her lips and exhibited a favorable outcome during the 5-year follow-up period.

## Discussion

EC is a very rare tumor in the SGs and is present in less than 1.1% of all epithelial SG neoplasia [3–5]. Most ECs have been known to be involved in major SGs including the parotid gland and do not often occur in minor SGs such as the lip mucosa. The final diagnosis of EC could be determined under routine microscopic examination where the malignancy is composed of an inner layer of ductal cells surrounded by a layer of clear myoepithelial cells, as seen in this presented case. Cytokeratin positivity in epithelial cells and clear myoepithelial cells can confirm the EC diagnosis [6], and this case also exhibited keratinized tumor epithelium floating in the lymphoid stroma only in the epithelial layer and not into the adjacent fibromuscular adipose tissue (Fig. 3). The differential diagnosis of EC composed of predominantly clear cells includes actinic cell adenocarcinoma, mucoepidermoid carcinoma, oncocytoma, and clear cell adenocarcinoma of MSGs [3–5]. There are no definite treatment guidelines for EC in the lips, but surgical excision with a wide resection margin could be recommended in cases involving major SG.

Comedo-type necrosis has been called as comedonecrosis, which is the central luminal inflammation with necrotic cells. This occurred usually in the breast cancer such as intraductal carcinoma or ductal carcinoma in situ; the breast duct is completely plugged by cancer cells. Solid or comedo growth patterns are high-grade ductal carcinoma in situ, if there is a corresponding variation in nuclei or evidence of necrosis. Like those of in our case (Fig. 2), high-grade nuclei of the tumor cells with frequent mitoses, abundant comedo-type necrosis, focal areas of concomitant squamous differentiation, consistent immunoreactivity for cytokeratins [7], and the basaloid tumor cells exhibited relatively undifferentiated cellular characteristics and undeveloped cell organelles in ultrastructural findings. The breast and salivary glands are both exocrine glands sharing similar morphologic features; thus, the salivary gland metaplasia could appear as a diffuse adenosis-like lesion in the breast, and the presence of benign salivary-type acini and ducts in the breast without accompanying salivary gland-type tumors had been reported [8].

**Fig. 4:**
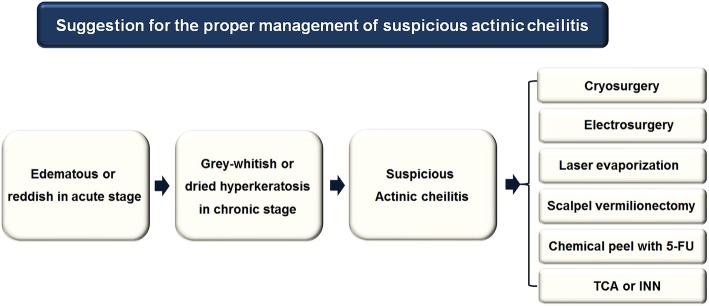
Schematic flowchart for the suggestion of the suspicious actinic cheilitis

AC occurs in patients over the age of 45, with a male predominance in a 10:1 ratio [9], and AC more often affects the lower lip versus the upper lip. AC is known to be caused by chronic sunlight exposure and ultraviolet radiation from outdoor lifestyles such as farming, sailing, fishing, windsurfing, mountaineering, and golfing, giving AC the name of sailor’s lip or farmer’s lip [1, 9]. Most AC patients complain of painless recurring symptoms including hard and dry sensations or cracking lip mucosal sensations. Diverse appearances of AC include edematous reddish in the acute stage and grey-whitish or dried hyperkeratotic wrinkled lesions in the chronic stage for several months or even years (Fig. 4) [10].

The differential diagnosis of AC includes actinic lichen planus (ALP), herpes simplex lesions, exfoliative cheilitis, contact cheilitis (CC), autoimmune blistering disease including pemphigus vulgaris (PV), lichenoid drug eruptions, cheilitis granulomatosa (CGM), cheilitis grandularis (CGL), and early carcinoma in situ (CIS) [11, 12]. ALP is a variant of OLF occurring in sunlight-exposed areas in patients with dark skin, and sunlight in lip LP has been suggested in lower lip involvement with male predominance. Typical histologic findings include lichenoid lymphocytic infiltrates with interface vacuolar changes and focal subepithelial blistering [12]. Exfoliative cheilitis exhibits hyperkeratosis and erythema in the histologic findings, which is associated with a history of factitial injury [9, 13]. CC is triggered by an allergy to toothpaste or beauty care products or an irritation to extremely hot, cold, and dry weather and characterized by scaling and erythema along the vermillion border of the lips with sloughing of the surface epithelium [11, 13]. PV is characterized by blisters, erosion, and an ulcer involving the lips and buccal mucosa. More than 85% of cases of oral PV are preceded by a cutaneous lesion [11, 14]. CGM is a rare condition manifesting as an episodic, non-tender enlargement of one or both lips and feels firm and nodular upon palpation [11, 15]. CGM could be considered with CGL as a potential predisposing factor for development of AC and SCC. CGL is a chronic inflammatory condition manifesting as MSG hypersecretion with ductal ectasia with swollen lips, nodular growth with everted margins, and ulceration [10, 11]. CGL is characterized by progressive enlargement and eversion of the lower labial mucosa that results in obliteration of the mucosal-vermilion interface [10, 11].

In this case, if the lower lip was diagnosed with premalignant AC or CGL, malignant transformation to EC could have been prevented with early management. If we know the clinical diagnosis entity as AC, wide V-shaped resection could be avoided. To prevent AC, chronic habits and irritation and sunlight exposure must be controlled and avoided. More active treatment options for AC include cryosurgery, electrosurgery, carbon dioxide laser vaporization, scalpel vermilionectomy, and chemical peel with 5-fluorouracil (5-FU), trichloroacetic acid (TCA), or imiquimod (INN) (Fig. 4).

Cryosurgery is the first treatment of choice for AC through application of liquid nitrogen to the lesion, with a cure rate greater than 96%. Electrosurgery is also a useful treatment method under local anesthesia, but could delay healing with scar formation of the adjacent tissue [1, 16]. Carbon dioxide laser vaporization with or without scalpel vermilionectomy could be used in broad or recurring AC and removes the entire vermillion border, leaving the underlying muscle intact. Although a linear scar may form, these techniques have been found to be effective and useful for surgical management of AC. Topical 5-FU is known to be effective against the minor form of AC by blocking DNA synthesis and having less of a negative effect on normal skin or lip mucosa. Complete remission has been reported in 50% of cases of AC after 2 to 4 weeks of application, with minimal scarring [1]. Topical TCA has been also used in 50% concentration form, as in the case of topical chemoablative application for genital warts or tattoo removal, but its limited reports have shown a low complete remission rate less than 30% [1, 17]. INN is one of the representative chemical peel agents for AC and promotes immune response induction of apoptosis of tumor cells. Complete remission of actinic keratosis has been seen in more than 45% of patients, but the long-term effects with the effective dose or duration has not been clearly identified in AC management [1].

## Conclusions

A very rare case of EC from an MSG in the lower lip mucosa exhibited marked ductal hyperplasia with well-localized tumor cells and abundant lymphoid tissue. The floating keratinized tumor epithelium may have originated from a previous AC lesion with a luminal sequestration of keratinized epithelium that was not properly managed over the course of 3 years. Accurate treatment of AC in its initial stage could be recommended to avoid further malignant transformation into SCC or EC, and superficial evaporation could be recommended instead of wide resection.

## Abbreviations

AC: Actinic cheilitis
ALP: Actinic lichen planus
CC: Contact cheilitis
CGL: Cheilitis grandularis
CGM: Cheilitis granulomatosa
CIS: Carcinoma in situ
CT: Computed tomography
EC: Epimyoepithelial carcinoma
EMC: Epithelial myoepithelial carcinoma
MRI: Magnetic resonance imaging
MSGs: Minor salivary glands
OLP: Oral lichen planus
PET-CT: Positron emission tomography-computed tomography
PV: Pemphigus vulgaris
SCC: Squamous cell carcinoma
SG: Salivary gland

## References

[1] Wikipedia, the free encyclopedia Center (2018). Wikipedia registered.

[2] Vieira RA, Minicucci EM, Marques ME, Marques SA (2012). Actinic cheilitis and squamous cell carcinoma of the lip: clinical, histopathological and immunogenetic aspects. An Bras Dermatol.

[3] Lokuhetty M, Premathilake I, Amarasinghe C (2011). Epimyoepithelial carcinoma - an uncommon salivary gland tumour. J Diag Pathol.

[4] Corio RL, Sciubba JJ, Brannon RB, Batsakis JG (1982). Epithelial myoepithelial carcinoma of intercalated duct origin. A cliniopathologic and ultrastructural assessment of sixteen cases. Oral Surg Oral Med Oral Pathol.

[5] Donath K, Seifert G, Schmitz R (1972). Diagnose and unltrastrukturdes tubularen speichelgangcarcinomas Epithelial myoepitheliales schaltstustuckcarcinom. Virchows Arch.

[6] Garcia NG, Oliveira DT, Lauris JR, Domingues MA, Minicucci EM, Soares C (2016). Loss of cytokeratin 10 indicates malignant transformation in actinic cheilitis. Clin Oral Investig.

[7] Li TJ, Zhang YX, Wen J, Cowan DF, Hart J, Xiao SY (2004). Basaloid squamous cell carcinoma of the esophagus with or without adenoid cystic features. Arch Pathol Lab Med.

[8] Jang EJ, Kang SH, Bae YK (2014). Basaloid ductal carcinoma in situ arising in salivary gland metaplasia of the breast: a case report. Int J Clin Exp Pathol.

[9] Jadotte YT, Schwartz RA (2012). Solar cheilosis. An ominous precursor Part II Diagnostic insights. J Am Acad Dermatol.

[10] Nico MM, Nakano de Melo J, Lourenço SV (2010). Cheilitis glandularis: a clinicopathological study in 22 patients. J Am Acad Dermatol.

[11] Muthukrishnan A, Bijai Kumar L (2017). Actinic cheilosis: early intervention prevents malignant transformation. BMJ Case Rep.

[12] Choi E, Tan KB, Chandran NS (2017). Isolated actinic lichen planus of the lower lip. Case Rep Dermatol.

[13] Picascia DD, Robinson JK (1987). Actinic cheilitis: a review of the etiology, differential diagnosis, and treatment. J Am Acad Dermatol.

[14] Bickle K, Roark TR, Hsu S (2002). Autoimmune bullous dermatoses: a review. Am Fam Physician.

[15] El-Hakim M, Chauvin P (2004). Orofacial granulomatosis presenting as persistent lip swelling: review of 6 new cases. J Oral Maxillofac Surg.

[16] Lubritz RR, Smolewski SA (1983). Cryosurgery cure rate of premalignant leukoplakia of the lower lip. J Dermatol Surg Oncol.

[17] Czerninski R, Zini A, Sgan-Cohen HD (2010). Lip cancer: incidence, trends, histology and survival: 1970-2006. Br J Dermatol.

